# A comparative analysis of myocardial strain and strain rate in cardiac computed tomography and magnetic resonance feature tracking

**DOI:** 10.1007/s11547-025-02060-5

**Published:** 2025-07-29

**Authors:** Irene Del-Canto, María P. López-Lereu, José V. Monmeneu, Alicia Maceira, David Moratal

**Affiliations:** 1 Departamento de Ingeniería Electrónica, Universitat de València, Valencia, Spain; 2 Centro de Investigación Biomédica en Red - Cardiovascular (CIBER-CV), Madrid, Spain; 3 Unidad de Imagen Cardíaca, ASCIRES, Valencia, Spain; 4 ASCIRES-UPV Joint Research Unit, Valencia, Spain; 5 Center for Biomaterials and Tissue Engineering, Universitat Politècnica de València, Camí de Vera, S/N, 46022 Valencia, Spain

**Keywords:** Cardiac magnetic resonance, Cardiac computed tomography, Feature tracking, Left ventricular function, Myocardial strain, Reproducibility

## Abstract

**Purpose:**

This study investigated the feasibility of feature tracking cardiac computed tomography (CCT)-derived LV global and regional strain and its agreement with feature tracking cardiac magnetic resonance (CMR)-derived measurements.

**Methods:**

CMR images and CCT images were acquired from 15 adult patients (50% women, mean age: 65 ± 9 years). Cardiac LV global and segmental circumferential and longitudinal strain and strain rate were assessed on CCT and CMR using feature tracking (FT) technology. Agreement between the measurements from two different modalities was evaluated using Bland–Altman analysis and intraclass correlation coefficient (ICC).

**Results:**

Strain/strain rate analysis could be accomplished in every subject with both techniques. Agreement between CMR-FT and CCT-FT was strong for global circumferential strain (ICC > 0.75), while other global strain features displayed moderate inter-technique correlation (ICC > 0.50). On Bland–Altman analysis, strain analysis derived from CCT-FT showed a lower measurement of LV global longitudinal strain and strain rate in comparison with CMR-FT (mean difference: 5.32% and 0.71 s^−1^, respectively). Additionally, reproducibility of segmental strain was better for myocardial and endocardial than epicardial walls.

**Conclusions:**

CCT-FT is a feasible technique, with good agreement with analogous CMR-FT-derived parameters, especially for global measurements, and could be an acceptable alternative for strain assessment.

**Graphical abstract:**

Overview for the comparative analysis of myocardial strain and strain rate assessed by computed tomography feature tracking and magnetic resonance feature tracking. CCT: cardiac computed tomography, CMR: cardiac magnetic resonance, FT: feature tracking, SSFP: steady‐state free precession, CS: circumferential strain, LS: longitudinal strain, SR-C: circumferential strain rate, SR-L: longitudinal strain rate, ICC: intraclass correlation coefficient 
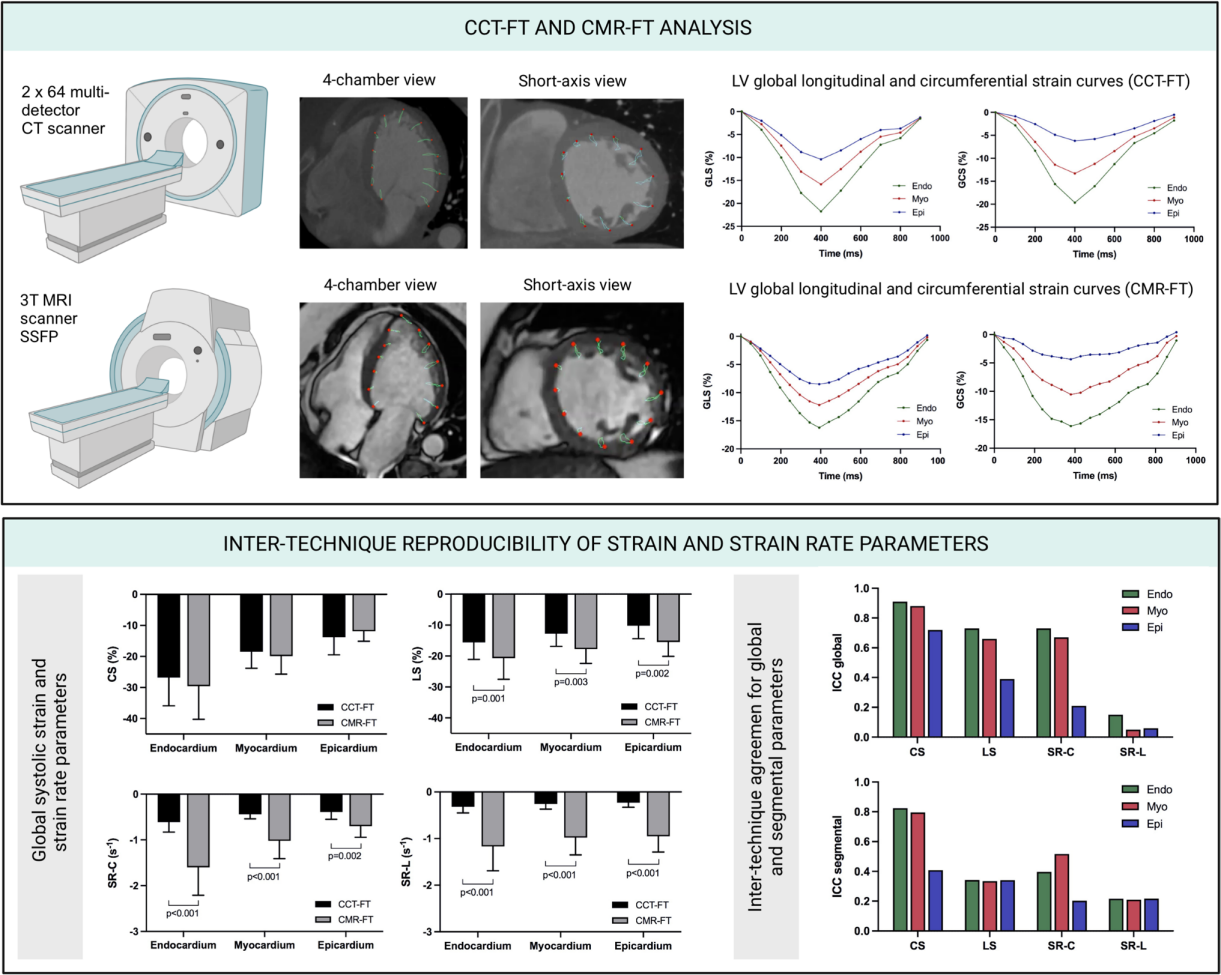

**Supplementary Information:**

The online version contains supplementary material available at 10.1007/s11547-025-02060-5.

## Introduction

Myocardial strain analysis has proved to be helpful in identifying and stratifying the risk of various cardiac conditions by evaluating regional and global myocardial function. In fact, strain and strain rate assessment have become more prevalent in clinical practice and research investigations [1, 2].

Cardiac magnetic resonance (CMR) is considered the reference standard for measurement of myocardial strain. A newly validated cardiac magnetic resonance feature tracking (CMR-FT) technique has been compared to myocardial tagging and is now widely accepted as the preferred tool for strain assessment in CMR [3]. The primary benefit of CMR-FT is its ability to be used on steady-state free precession (SSFP) cine images commonly utilized in clinical settings without needing any extra image capture, allowing a fast and less reliant on observer evaluation of strain [4–6].

Cardiac computed tomography (CCT) is the most recent and quickly expanding method for non-invasive heart imaging. Although CCT studies are often used to evaluate the coronary arteries, they can also be adjusted to obtain functional data for the left and right ventricles as well [7–9], with close correlation with CMR [10]. Recently, there have been new advancements in the development of novel techniques for assessing regional myocardial function using CCT, as the feature tracking algorithm applied to cardiac computed tomography (CCT-FT) images to evaluate myocardial strain [11–14]. However, there is limited information concerning the assessment of strain and strain rate by CCT-FT and its correlation with CMR-FT-based quantification. [15, 16]

In this current research, we aimed to assess the agreement between feature tracking CT-derived and feature tracking MR-derived global and segmental ventricular strain measurements.

## Materials and methods

### Study group

Fifteen adult patients were included (Table 1) who had been referred to our unit for both CMR and helical CCT studies in a time interval of less than 6 months. The exclusion criteria were: recurrent MIs, severe clinical instability or cardiac surgery, contraindication to CMR, and insufficient CMR study quality. The study was carried out in accordance with the guidelines and regulations of the Declaration of Helsinki (2008) and was approved by the Medical Ethical Committee of our hospital. All participants provided written informed consent.

**Table 1:** Baseline and CMR characteristics of the studied population

	Subjects (n = 15)
Age (years)	65 ± 9
Female/male gender	5/10
Body mass index (kg/m^2^)	24 ± 2
Heart rate on admission (beats/min)	63 ± 12
Heart rate during CMR scan (beats/min)	67 ± 10
Heart rate during CCT scan (beats/min)	66 ± 9
Systolic blood pressure (mmHg)	129 ± 23
Diastolic blood pressure (mmHg)	67 ± 8
Hypertension (%)	33
Diabetes mellitus (%)	26
Dyslipidemia (%)	40
Tobacco use (%)	33
History of arrhythmias (%)	7
*Agatston score*	
0–100	73
101–300	0
> 300	20
CAD (stenosis > 50%)	20
*LV volumes and LV function*	
LV ejection fraction (%)	56 ± 18
LVED volume index (ml/m^2^)	91 ± 41
LVES volume index (ml/m^2^)	53 ± 59
LV mass index (g/m^2^)	80 ± 32
*RV volumes and RV function*	
RV ejection fraction (%)	61 ± 5
RVED volume index (ml/m^2^)	77 ± 25
RVES volume index (ml/m^2^)	30 ± 12
*LV pattern*	
Normal (%)	73
Concentric remodeling (%)	0
Eccentric hypertrophy (%)	13
Concentric hypertrophy (%)	13
Necrotic pattern on late gadolinium enhancement (%)	0
Effective radiation dose (mSv)	10.59 ± 1.48

Values are mean ± SD or %. *LVED* left ventricular end-diastole, *LVES* left ventricular end-systole

### CMR protocol

CMR was performed with one 3 T scanner (Vida, Siemens, Erlangen, Germany, and Signa, GE, Milwaukee, USA) using retrospective ECG triggering. Steady-state free precession (SSFP) end-expiratory breath-hold cines were obtained in both vertical and horizontal long-axis planes, following by continuous short-axis cines from the atrioventricular (AV) ring to the apex and acquisition of 2-, 3, and 4-chamber view cines. Slice thickness was 8 mm with a gap of 2 mm between slices when necessary. Normally, each cine sequence contained 40 phases with an average temporal resolution of 21.5 ms. Common sequence parameters consisted of a repetition time / echo time (TR/TE) of 3.3/1.4 ms, flip angle of 48º, matrix size of 256 × 230, field of view (FoV) of 370 × 296, and acquisition time of 10 s.

### CMR image analysis

**Fig. 1:**
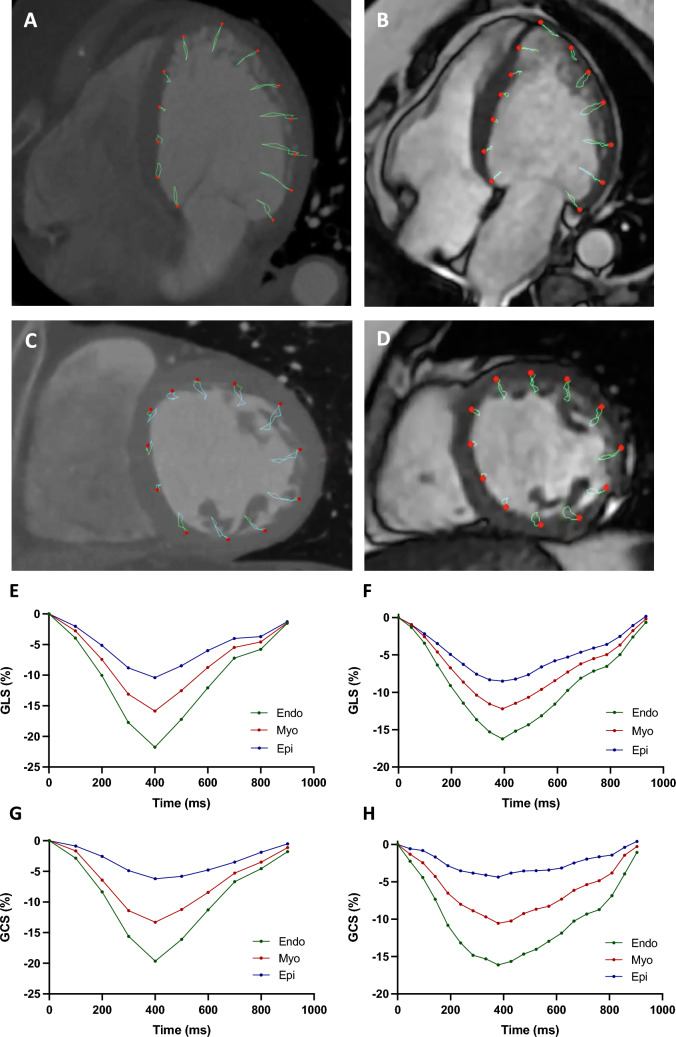
Assessment of LV global longitudinal and circumferential strain using CMR-FT and CCT-FT analysis. Left ventricular 4-chamber view (**a**, **b**) and short-axis mid-ventricular view (**c**, **d**) for CCT-FT (left) and CMR-FT (right). Representation of LV global longitudinal and circumferential strain (GLS, GCS) curves using CCT-FT (**e**, **g**) and CMR-FT (**f**, **h**)

LV endocardial and epicardial contours were semi-automatically outlined in the long-axis and short-axis views at end-diastole and end-systole using Medis QMass software (version 8.1; Medis Medical Imaging Systems, Leiden, The Netherlands), and manual adjustment was performed as needed. Subsequently, 2D cine images were analyzed using commercial feature tracking (FT) software package (Medis QStrain 2.0; Medis Medical Imaging Systems, Leiden, The Netherlands). For segmental analysis, LV short-axis and long-axis views were divided based to the 16-segment model of the American Heart Association (AHA) [17]. Peak longitudinal strain and strain rate were calculated by tracking long-axis cine images; peak circumferential strain and strain rate were obtained by tracking short-axis cine images (Fig. 1).

### CCT protocol

Contrast-enhanced cardiac helical scans with retrospective ECG-gating were conducted using one of two 64-slice multi-detector computed tomography scanners (Discovery 750HD, GE, Milwaukee, WI). The acquisition was synchronized with the ECG, and the standard study protocol was utilized: tube voltage 100–120 kV, tube current 200–450 mAs with ECG dose modulation. Rotation time 350 ms, slice collimation of 0.625 × 0.625 mm, with a temporal resolution of 165 ms, slice thickness of 0.625 mm, reconstruction increment of 0.625 mm, standard reconstruction kernel, and 40 mm coverage per rotation, enable the capture of a volume containing the heart in an average breath-hold time of 6 s.

Nonionic iodine contrast (Iomeron 400, Bracco, Italy) was injected through the antecubital vein, with the volume tailored to the patient’s weight, at an infusion rate of 5 ml/s. The acquisition started when the contrast reached the ascending aorta. Typical volume injected was 70 ml. Images were acquired from the tracheal carina to the diaphragm in a cranio-caudal direction. Administration of intravenous betablocker was not used. The effective radiation dose of enhanced CCT was calculated by multiplying DLP (dose–length product) by 0.014 mSv/(mGy cm) [18].

### CCT image analysis

Three-dimensional (3D) datasets were analyzed offline using Medis 3D View (version 3.2; Medis Medical Imaging Systems, Leiden, The Netherlands) to create two-dimensional (2D) cine images of three long-axis views (2-, 3-, 4-chamber), three short-axis views (basal, mid, apical), and a stack of short-axis slices with a thickness of 0.75 mm. Images were generated with a temporal resolution of 10 phases in each cardiac cycle, spanning from early systole to end-diastole in increments of 10% [12]. Special attention was paid to ensure that 2D cardiac CT reconstructions accurately aligned with the anatomical positions of the images utilized for CMR analysis. Contour tracing and feature tracking analysis were carried out analogously to CMR image analysis and with the same software packages as referred above. Briefly, endocardial and epicardial contours of the LV were semi-automatically outlined in the long-axis and short-axis views at end-diastole and end-systole with manual correction done when necessary. The reconstructed 2D cine images were then analyzed using the same feature tracking (FT) software package. For segmental analysis, LV short-axis and long-axis views were divided according to the 16-segment model of the American Heart Association (AHA) [17]. Peak longitudinal strain and strain rate were calculated by tracking long-axis cine images; peak circumferential strain and strain rate were obtained by tracking short-axis cine images (Fig. 1).

### Assessment of reproducibility

To assess intra-observer reproducibility for the measurement of CMR-FT and CCT-FT global and regional strain, the same 30 scans were reanalyzed by the observer with a 1-month temporal separation between analyses.

### Statistical analysis

Statistical analysis was performed using SPSSv.24.0 (IBM, Armonk, NY), and statistical significance was considered when *p* < 0.05. Continuous variables were expressed as the mean ± standard deviation, according to normality of distribution, assessed by Shapiro–Wilk test. Categorical data are presented as percentages.

Strain measurements from two techniques were compared using paired samples t-test. A Bland–Altman analysis was used to visually represent the range of values obtained through both techniques, showing the mean bias and 95% limits of agreement [19]. Inter-technique reproducibility was evaluated using intraclass correlation coefficient (ICC) for absolute agreement. Agreement levels were specified based on prior research [20]: excellent for ICC > 0.90, good for ICC 0.75–0.90, moderate for ICC 0.50–0.75, and poor for ICC < 0.50. Analyses achieved for global measurements were repeated for regional measurements. Additionally, to provide a comprehensive evaluation of inter-technique reproducibility based on coronary pathology, the agreement between the two techniques was assessed separately in segments corresponding to affected coronary artery territories and in those considered healthy.

Intra-observer reliability of measurements was assessed using intraclass correlation coefficient (ICC) with an absolute agreement two-way random effect mode. ICC estimates and their 95% confident intervals (CI) were reported.

## Results

The baseline characteristics and CMR and CCT data concerning the subjects under study are presented in Table 1. Participants were adults of middle age and both genders, average 64 ± 9 years. Concerning cardiovascular risk factors in the patient group, 33% were hypertensive, 47% had dyslipidemia, 20% were smokers or former smokers, and 13% had diabetes. Additionally, 10 (66%) patients showed alterations in functional and structural LV characteristics: 4 (26%) patients had increased LVED volume index, 6 (40%) patients had depressed LVEF, and 5 (33%) presented increased LV mass index or alterations in LV geometric patterns. Regarding the right ventricle (RV), 2 patients (13%) showed alterations which in both cases were mildly increased RVED volume index. The average heart rate during CCT and CMR scanning was 66 ± 9 beats per minute (bpm) and 67 ± 10 bpm, respectively. Three subjects (20%) had a heart rate greater than 70 bpm. No evidence of cardiac arrhythmias was observed in most patients (93%). The mean effective radiation dose of CCT was 10.59 ± 1.48 mSv.

### Feasibility of strain and strain rate measurements

Imaging quality was classified as excellent in 80% of patients and good to moderate in 20% of patients. CMR-FT and CCT-FT analysis was successful in every scan, showing a feasibility rate of 100%. Tracking quality was adequate in every segment. LV strain and strain rate were analyzed in every segment of all patients, allowing for direct comparisons between modalities in each case.

### Inter-technique variability of global strain and strain rate parameters

Table 2 shows peak global systolic circumferential and longitudinal strain (CS, LS) and strain rate (SR-C, SR-L) average values for myocardium, endocardium and epicardium walls assessed by CMR-FT and CCT-FT analysis. Global circumferential strain measurements did not show significant inter-technique differences for myocardium, endocardium, and epicardium (*p* = 0.191, *p* = 0.060, and *p* = 0.318, respectively). Conversely, global longitudinal strain was inferior in CCT-FT vs. CMR-FT for myocardium (− 12.8 ± 4.1 vs. − 17.8 ± 4.6%, *p* < 0.01), endocardium (− 15.6 ± 5.5 vs. − 20.7 ± 6.8%, *p* < 0.01), and epicardium (− 10.2 ± 4.2 vs. − 15.5 ± 4.6%, *p* < 0.01). Similarly, strain rate parameters (circumferential and longitudinal) assessed by CCT-FT showed less values than those assessed by CMR-FT (*p* < 0.001).

**Table 2:** Global systolic strain and strain rate values in myocardium, endocardium and epicardium walls

Parameter	Wall	CCT-FT	CMR-FT	*p *value
CS (%)	Myocardium	− 18.5 ± 5.3	− 19.9 ± 5.8	0.191
	Endocardium	− 26.8 ± 9.1	− 29.6 ± 10.7	0.060
	Epicardium	− 13.8 ± 5.7	− 11.9 ± 3.2	0.076
LS (%)	Myocardium	− 12.8 ± 4.1	− 17.8 ± 4.6	0.001
	Endocardium	− 15.6 ± 5.5	− 20.7 ± 6.8	0.003
	Epicardium	− 10.2 ± 4.2	− 15.5 ± 4.6	0.002
SR-C (s^−1^)	Myocardium	− 0.44 ± 0.10	− 1.02 ± 0.39	0.000
	Endocardium	− 0.61 ± 0.22	− 1.60 ± 0.61	0.000
	Epicardium	− 0.39 ± 0.16	− 0.70 ± 0.24	0.002
SR-L (s^−1^)	Myocardium	− 0.26 ± 0.11	− 0.98 ± 0.37	0.000
	Endocardium	− 0.32 ± 0.13	− 1.17 ± 0.52	0.000
	Epicardium	− 0.23 ± 0.10	− 0.95 ± 0.34	0.000

*CS* circumferential strain, *LS* longitudinal strain, *SR* strain rate, *CCT-FT* cardiac computed tomography feature tracking, *CMR-FT* cardiac magnetic resonance feature tracking

**Fig. 2:**
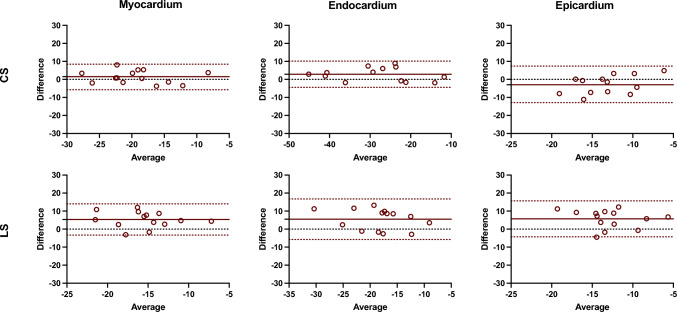
Bland–Altman analysis of the global circumferential and longitudinal strain assessment between CMR-FT and CCT-FT. The horizontal red solid line describes the mean; the 2 red dash lines describe the upper and lower limits of agreement. CS: circumferential strain, LS: longitudinal strain

**Fig. 3:**
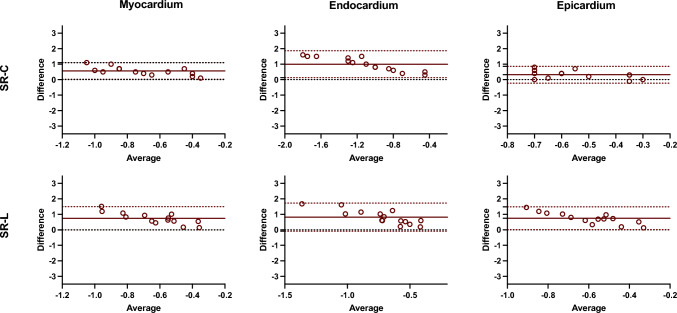
Bland–Altman analysis of the global circumferential and longitudinal strain rate assessment between CMR-FT and CCT-FT. The horizontal red solid line describes the mean; the 2 red dash lines describe the upper and lower limits of agreement. SR-C: circumferential strain rate, SR-L: longitudinal strain rate

Figures 2 and 3 show Bland–Altman plots comparing the different techniques for global strain and strain rate measurements, respectively. The average inter-technique absolute bias was 1.34 ± 3.62%, 2.84 ± 3.72%, and − 2.79 ± 5.16% for CS measures and 5.32 ± 4.41%, 5.48 ± 5.74%, and 5.67 ± 5.10% for LS measures, in myocardium, endocardium, and epicardium, respectively (Table 3; Fig. 2) In relation to the inter-technique agreement, the intraclass correlation coefficient (ICC) values were good to excellent for the circumferential global strain measurements in the myocardium and endocardium, and moderate in epicardium. Similarly, myocardial and endocardial longitudinal strain showed moderate reproducibility, whereas epicardial LS had poor reproducibility (Table 3).

**Table 3:** Inter-technique reproducibility for global systolic strain and strain rate parameters

Parameter	Wall	Mean bias ± SD	LOA	*p* value	ICC (95% CI)
CS (%)	Myocardium	1.34 ± 3.62	− 5.75, 8.43	0.000	0.88 (0.63, 0.96)
	Endocardium	2.84 ± 3.72	− 4.46, 10.13	0.000	0.91 (0.59, 0.98)
	Epicardium	− 2.79 ± 5.16	− 12.91, 7.33	0.190	0.72 (0.05, 0.85)
LS (%)	Myocardium	5.32 ± 4.41	− 3.33, 13.97	0.030	0.66 (− 0.05, 0.89)
	Endocardium	5.48 ± 5.74	− 5.77, 16.7	0.013	0.73 (0.15, 0.91)
	Epicardium	5.67 ± 5.10	− 4.31, 15.66	0.190	0.39 (− 0.89, 0.81)
SR-C (s^−1^)	Myocardium	0.56 ± 0.27	0.03, 1.09	0.025	0.67 (0.00, 0.89)
	Endocardium	0.99 ± 0.44	0.12, 1.86	0.011	0.73 (0.18, 0.91)
	Epicardium	0.32 ± 0.28	− 0.23, 0.86	0.347	0.21 (− 1.60, 0.76)
SR-L (s^−1^)	Myocardium	0.74 ± 0.38	− 0.01, 1.50	0.466	0.05 (− 1.97, 0.69)
	Endocardium	0.82 ± 0.46	− 0.09, 1.72	0.386	0.15 (− 1.55, 0.71)
	Epicardium	0.75 ± 0.38	0.01, 1.49	0.538	0.06 (− 2.29, 0.66)

*CS* circumferential strain, *LS* longitudinal strain, *SR* strain rate, *LOA* limits of agreement, *ICC* intraclass correlation coefficient, *CI* confidence intervals

Regarding SR measures, overall inter-technique absolute bias in ventricular myocardium was 0.56 ± 0.27 s^−1^ for SR-C and 0.71 ± 0.52 s^−1^ for SR-L. In the endocardium, the inter-technique bias was 0.99 ± 0.44 s^−1^ for SR-C and 0.89 ± 0.69 s^−1^ for SR-L, and in the epicardium, a value of − 0.32 ± 0.28 s^−1^ was obtained for SR-C and 0.66 ± 0.60 s^−1^ for SR-L (Table 3; Fig. 3). Additionally, the intraclass correlation coefficient (ICC) values were moderate for the SR-C measurements in the myocardium and endocardium (Table 3). However, the rest of the strain rate parameters analyzed presented ICC values lower than 0.40, thus considering low reproducibility.

### Inter-technique variability of segmental strain and strain rate parameters

**Fig. 4:**
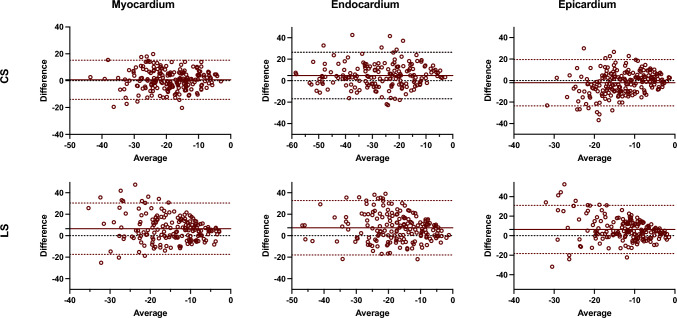
Bland–Altman analysis of the segmental circumferential and longitudinal strain assessment between CMR-FT and CCT-FT. The horizontal red solid line describes the mean; the 2 red dash lines describe the upper and lower limits of agreement. CS: circumferential strain, LS: longitudinal strain

**Fig. 5:**
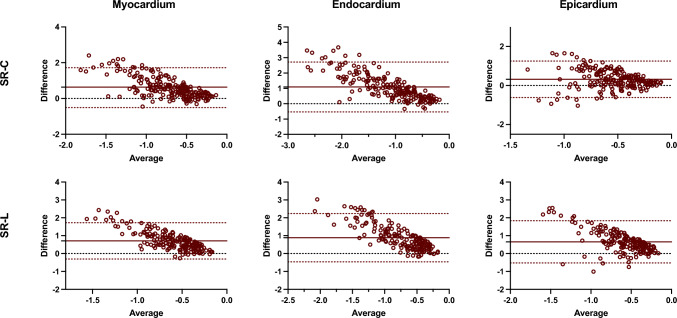
Bland–Altman analysis of the segmental circumferential and longitudinal strain rate assessment between CMR-FT and CCT-FT. The horizontal red solid line describes the mean; the 2 red dash lines describe the upper and lower limits of agreement. SR-C: circumferential strain rate, SR-L: longitudinal strain rate

**Fig. 6:**
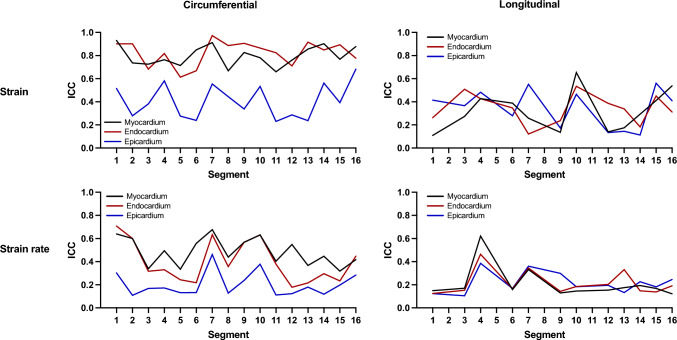
Inter-technique intraclass correlation coefficients for segmental strain and strain rate parameters (myocardium, endocardium, epicardium). CS: circumferential strain, LS: longitudinal strain, SR-C: circumferential strain rate, SR-L: longitudinal strain rate, ICC: intraclass correlation coefficient

Segmental reproducibility was also evaluated for segmental peak systolic CS, LS, SR-C, and SR-L, respectively. Figures 4 and 5 show Bland–Altman plots comparing the different techniques for segmental strain and strain rate measurements, respectively. For all the subjects and segments included, mean inter-technique absolute bias was 0.63 ± 7.43%, 4.77 ± 11.05%, and − 2.03 ± 11.01% for CS values and 6.49 ± 12.19%, 7.39 ± 12.93%, and 6.36 ± 12.58% for LS values, in myocardium, endocardium, and epicardium, respectively (Fig. 4). Additionally, Fig. 6 plots inter-technique ICC per myocardial segments. Reproducibility of circumferential strain was moderated to excellent for myocardial and endocardial segments (0.61–0.97), whereas epicardial segments showed fair agreement (average 0.41). Conversely, consistency of longitudinal strain was poor for all segments in three ventricular walls.

With respect to SR measurements, average inter-technique absolute bias was 0.56 ± 0.27 s^−1^, 0.99 ± 0.44 s^−1^, and 0.32 ± 0.28 s^−1^ for circumferential values and 0.74 ± 0.38 s^−1^, 0.82 ± 0.46 s^−1^, and 0.75 ± 0.38 s^−1^ for longitudinal values, in myocardium, endocardium, and epicardium, respectively (Fig. 5). Furthermore, circumferential strain rate showed moderate agreement for the myocardial segments (average 0.52) and low to moderate for the endocardial segments (average 0.40), whereas epicardial segments showed the lower concordance values. Similar to the concordance values of strain, reproducibility of longitudinal strain rate was poor for all segments (Fig. 6). Overall, strain rate provided less reliable outcomes than strain measurements.

### Inter-technique reproducibility based on coronary pathology

In the analysis of inter-technique reproducibility across myocardial layers (endocardium, mid-myocardium, and epicardium), no significant differences were observed between segments with coronary artery disease (CAD) and those without (non-CAD), for either circumferential strain (CS) or longitudinal strain (LS) (Table 4). Similarly, no significant differences were observed for strain rate parameters (SR-CS and SR-LS) between CAD and non-CAD segments, at any of the myocardial walls.

**Table 4:** Inter-technique reproducibility for systolic strain and strain rate parameters in CAD and non-CAD segments

Parameter	Wall	CAD segments ICC (95% CI)	Non-CAD segments ICC (95% CI)	*p* value (z-test)
CS (%)	Myocardium	0.82 (0.52, 0.93)	0.80 (0.73, 0.85)	0.810
	Endocardium	0.86 (0.59, 0.95)	0.89 (0.83, 0.93)	0.670
	Epicardium	0.63 (0.32, 0.80)	0.71 (0.61, 0.78)	0.462
LS (%)	Myocardium	0.69 (0.17, 0.89)	0.65 (0.46, 0.76)	0.783
	Endocardium	0.74 (0.32, 0.90)	0.72 (0.56, 0.82)	0.887
	Epicardium	0.41 (− 0.64, 0.80)	0.39 (0.15, 0.56)	0.866
SR-C (s^−1^)	Myocardium	0.64 (0.06, 0.87)	0.61 (− 0.11, 0.83)	0.786
	Endocardium	0.76 (0.06, 0.93)	0.73 (0.22, 0.87)	0.759
	Epicardium	0.26 (− 0.72, 0.72)	0.21 (− 0.04, 0.40)	0.767
SR-L (s^−1^)	Myocardium	0.07 (− 0.19, 0.43)	0.07 (− 0.05, 0.20)	1.000
	Endocardium	0.17 (− 0.35, 0.62)	0.16 (− 0.12, 0.39)	0.967
	Epicardium	0.06 (− 0.33, 0.51)	0.07 (− 0.15, 0.22)	0.968

*CS* circumferential strain *LS* longitudinal strain, *SR* strain rate, *CAD* coronary artery disease, *ICC* intraclass correlation coefficient, *CI* confidence intervals

### Intra-observer reproducibility

Intra-observer reproducibility was good to excellent for all analyzed global strain measures. Regarding measurements of global CMR-FT strain, ICC values (and their 95% CI) were 0.984 (0.933–0.995), 0.954 (0.569–0.989), 0.946 (0.442–0.987), and 0.948 (0.497–0.974) for CS, LS, SR-CS, and SR-LS, respectively. For measurements of global CCT-FT strain, ICC values were 0.975 (0.909–0.993), 0.945 (0.454–0.987), 0.891 (0.659–0.966), and 0.930 (0.208–0.984) for CS, LS, SR-CS, and SR-LS, respectively. Additionally, although the agreement on measurement of both strain and strain rate was moderated to excellent in all segments, it was quite lower for CCT-FT technique, especially in inferior and septal regions (Table S1).

## Discussion

### Main findings

In the present work, we quantified the contractile function of the LV with CCT feature tracking strain and calculated the agreement of this technique with the reference standard CMR feature tracking for assessment of global and regional strain. The findings can be summarized as follows: (a) CCT-FT was successfully performed in all the subjects and all myocardial segments, allowing the evaluation not only of global LV, but also of segmental LV strain and strain rate; (b) agreement of CCT-FT to CMR-FT was excellent for global and segmental CS and moderate for global and segmental LS, (c) strain rate derived from CCT-FT has a moderate correlation with CMR-FT, and (d) reproducibility was highest for both strain and strain rate at myocardial and endocardial walls.

In the last decade, cardiac MRI has been shown to be feasible for measuring myocardial strain in order to quantify global and regional cardiac function by evaluating myocardial deformation in a range of heart conditions [1]. However, cardiac CT is frequently used in clinical practice as a non-invasive and beneficial tool for evaluating coronary artery stenosis and plaque features [21]. In recent years, multiple techniques to measure myocardial strain parameters from cardiac CT images have been developed, and there are several studies that have shown the potential of this imaging modality in facilitating the quantitative assessment of myocardial strain [9, 12, 21]. Nevertheless, to our knowledge, this is the first study that has compared CCT-FT and CMR-FT in the assessment of global and segmental strain and strain rate parameters.

As previously mentioned, in this research, we utilized a commercially available feature tracking software to calculate strain from cardiac CT and MR datasets. Strain and strain rate analysis with CCT-FT and CMR-FT was feasible in all the subjects included. Nevertheless, not all strain features had a strong inter-modality reproducibility. It is not surprising that the agreement between CMR-FT and CCT-FT assessment was highest for parameters that were highly reproducible in both modalities, as circumferential and longitudinal strain [6, 12]. In fact, previous literature has shown that CS would be the most reproducible measure [6, 11, 22, 23]. Additionally, there has been reliable agreement regarding myocardial function between cardiac CT images and other imaging modalities. Previously, in a study by Miskinitye et al*.*, CCT-FT-derived global strain parameters (GLS and GCS) presented good correlation with CMR-FT, where Bland–Altman analysis showed comparable wide limits of agreement for GCS and GLS but a higher inter-technique bias for GLS [12]. Our results agree these findings. In a study of the same year, Ammon et al*.* showed a close correlation (*r* = − 0.8) between 3D principal strain and speckle tracking echocardiography derived GLS in a cohort of severe aortic stenosis patients [24]. Recently, Wang et al*.* observed close correlations between CMR-FT and CCT-FT regarding LV global strain (GCS, *r* = 0.86; GLS, *r* = 0.79) [23]. Moreover, in the greatest study to date (*n* = 123), Fukui et al*.* [16] demonstrated moderate correlation between CCT and transthoracic echocardiography. The lower temporal resolution of CCT compared to CMR could explain the greater inter-modality bias in longitudinal strain, as peak strain values are less likely to be captured in sequences with a lower number of frames [14]. Longitudinal strain involves more complex motion patterns than circumferential strain and therefore requires finer temporal resolution to be accurately assessed. In contrast, circumferential strain is less affected by lower frame rates, as motion in the circumferential direction is more continuous and less abrupt [25]. Moreover, the assessment of longitudinal parameters typically requires accurate delineation between the papillary muscles and the endocardial border in long-axis views, which can be more technically challenging [14].

Conversely, we observed that agreement was limited for LV strain rate measurements. This agrees with other reports [6, 25, 26]. Strain rate measurements were greatly influenced by frame rate in our research, where cine sequences and CT images were captured at 40 and 10 frames per cardiac cycle, respectively. These differences in temporal resolution may partly explain the lower reproducibility observed in strain rate measurements, which are particularly sensitive to the number of frames acquired per cardiac cycle [25, 27]. Additionally, the time of peak strain may differ between MR and CT techniques, and there are methodological discrepancies in strain assessment between both imaging modalities. In fact, the performance of feature tracking software may vary between CCT and CMR, and differences in contour initialization and tracking methodology could further contribute to the underestimation of both strain and strain rate. Finally, it should be noted that CMR sequences CMR sequences enable cine imaging with continuous myocardial motion tracking, whereas CCT relies on a stepwise acquisition approach, which represents an intrinsic limitation of the technique.

Overall, we observed better correlation between techniques for global parameters than for segmental parameters. CCT-FT consistently underestimated segmental strain variables (circumferential and longitudinal) and GLS compared to CMR-FT, whereas no differences were observed between techniques in GCS assessment. Previous studies have reported a similar underestimation of LV GLS when comparing CCT-derived strain to speckle tracking echocardiography [11, 13, 24] or to CMR tagging and CMR feature tracking [12, 23]. We hypothesize that the reduced temporal resolution of CCT, in comparison with CMR, results in bias between imaging modalities, as peak strain may not be as easily detected in sequences with fewer frames [12, 14, 25, 27, 28]. In a previous study, Chen et al*.* [27] observed that using a narrow reconstruction increment (5% R-R interval) resulted in significantly higher CCT-FT left ventricular strain values compared to a broader increment (10% R-R). Similarly, Backhaus et al*.* [25] demonstrated that increasing temporal resolution was associated with higher absolute strain and strain rate (SR) values, emphasizing that strain measurements require only moderate temporal resolution, whereas SR assessments benefit more directly from higher frame rates. Therefore, the lower acquisition frame rate of CCT compared to CMR could contribute to the lower reliability of CCT-derived strain parameters.

Regarding the inter-technique reproducibility of regional strain, there are few studies in the literature that quantify this parameter using CMR-FT or CCT-FT. Previous reports have demonstrated that CMR-FT- and CCT-FT-derived segmental CS showed better reliability than LS [6, 26, 29–32], which may explain the greater inter-modality agreement observed for myocardial circumferential values. However, for segmental longitudinal strain, reproducibility varied notably among different myocardial segments, ranging from poor to good, and was consistently lower than that of global strain. This relatively low and heterogeneous reproducibility of segmental LS may be attributed to the through-plane and twisting motion, which can result in the loss of tracked features and lead to low strain value even in segments without dyskinesia [30]. Additionally, it is worth noting that interobserver agreement tends to be higher for CMR-derived strain parameters compared to those derived from CCT [23, 33].

Concerning the evaluation of inter-technique variability in CAD and non-CAD segments, the results have shown that the agreement was not substantially affected by the presence of coronary pathology. These findings suggest that inter-technique reproducibility is preserved regardless of the underlying coronary status, supporting the robustness of strain measurements in both diseased and healthy myocardial tissue.

Finally, all strain and strain rate parameters provided better inter-technique reliability in myocardial and endocardial regions than epicardial wall. These differences in reproducibility are probably due to the greater accuracy in the delimitation of the endocardial contour (endocardial-blood border), with respect to that of the epicardial contour (epicardium-pericardium border).

One of the main challenges in comparing CMR-FT and CCT-FT lies in the intrinsic variability related to post-processing techniques. Recently, the development of advanced image analysis methods and the integration of deep learning techniques are contributing to improve the accuracy of CCT-FT. Deep learning-based models, such as *StrainNet*, have shown improved performance in assessing myocardial displacement from CT cines, even under limited temporal resolution [34, 35]. In addition, motion correction algorithms and automated segmentation approaches enhance the robustness of myocardial deformation analysis, reducing interobserver variability and improving reproducibility [36, 37]. Although these innovations are still undergoing clinical validation, they may help reduce the gap between CCT-FT and more established techniques like CMR-FT, particularly in settings where cardiac MRI is contraindicated or unavailable.

### Clinical implications

This study has shown that quantitative assessment of LV strain is feasible using CCT feature tracking, with good intra-observer agreement. This technique may represent a valuable alternative for evaluating myocardial deformation in specific patients for whom other techniques—such as speckle tracking echocardiography or MRI—are not feasible. This includes patients with inadequate acoustic windows, severe claustrophobia, or cardiac implantable electronic devices, the prevalence of which continues to rise in clinical practice. The presence of these devices often limits the applicability of CMR due to potential imaging artifacts or the inability to safely adapt device settings, highlighting the need for alternative imaging modalities.

Looking ahead, as the availability of next-generation systems continues to expand and radiation exposure is progressively reduced (3–4 mSv in dual-source CT scanner with volumetric acquisition) [38, 39], CCT-FT may become more routinely integrated as a complementary tool in cardiac CT studies performed with retrospective acquisition. In particular, the ability to accurately assess myocardial strain by CCT-FT plays a significant role in the clinical care of patients in whom GLS measurement may lead to changes in their treatment (such as in the case of cardiotoxicity due to chemotherapy). Additionally, its application in patients with ischemic cardiomyopathy undergoing non-invasive coronary angiography with CT could help assess the viability of dependent segments of diseased coronary arteries. By integrating coronary angiography with assessment of regional cardiac function, CCT-FT could offer valuable insights to support more precise treatment decision and improved risk evaluation in this patient population. Finally, the fast acquisition of CT images and the similarity of the strain quantification method with MR-based technique may facilitate its integration into regular clinical practice.

### Study limitations

Several limitations of our study should be mentioned. First, the relatively small sample size could limit the generalizability of the findings. Nevertheless, the cohort was relatively homogeneous, which may have contributed to reduced inter-patient variability and supported the consistency of the observed results. Second, this research has a retrospective design, with the data collected from a limited number of patients from a single institution. Third, to assess strain using CCT-FT, it is required to perform multiphase reconstructions which involve a retrospective ECG-gated acquisition, and a greater radiation exposure compared to typical CT protocols (overall radiation dose of 10 mSv). This limitation is particularly relevant when compared to the advantages of CMR, which allows myocardial assessment without exposure to ionizing radiation, making it safer option for serial evaluations. However, it should be mentioned that CCT enables simultaneous coronary and myocardial assessment in a faster single acquisition, making it useful in emergency settings and for routine clinical implementation. Finally, the standard values for CCT-FT derived strain are not established and require comparison to the reference modality CMR-FT in larger cohorts.

## Conclusions

CCT-FT is a feasible and promising technique for quantitative assessment of LV deformation, with good agreement with analogous CMR-FT-derived parameters, especially for global measurements. Despite some limitations, mainly related to the temporal resolution of CCT acquisitions, CCT-FT could be an acceptable alternative for other methods of strain assessment when these are not available.

## Supplementary Information

Below is the link to the electronic supplementary material.

Supplementary file1 (DOCX 31 kb)
