# Supplementary material for: A comparative analysis of myocardial strain and strain rate in cardiac computed tomography and magnetic resonance feature tracking
Source: Radiol Med. 2025 Jul 29;130(8):1158–71. doi: 10.1007/s11547-025-02060-5 (PMC12367947; doi:10.1007/s11547-025-02060-5)
Supplement: Supplementary file 1 — Supplementary file1 (DOCX 31 kb) [file 11547_2025_2060_MOESM1_ESM.docx]

**Supplementary Information**

**Table S1.** Intra-observer reproducibility for segmental systolic strain and strain rate

| Parameter | Segment | CMR-FT |  | CCT-FT |
| --- | --- | --- | --- | --- |
|  |  | ICC (95% CI) |  | ICC (95% CI) |
|  |  |  |  |  |
| CS | 1. Basal anterior | 0.992 (0.974-0.998) |  | 0.814 (0.478-0.942) |
|  | 2. Basal anteroseptal | 0.989 (0.954-0.998) |  | 0.840 (0.538-0.951) |
|  | 3. Basal inferoseptal | 0.950 (0.909-0.972) |  | 0.663 (0.177-0.889) |
|  | 4. Basal inferior | 0.974 (0.935-0.980) |  | 0.777 (0.395-0.930) |
|  | 5. Basal inferolateral | 0.987 (0.975-0.991) |  | 0.865 (0.601-0.959) |
|  | 6. Basal anterolateral | 0.981 (0.942-0.994) |  | 0.764 (0.368-0.925) |
|  | 7. Mid anterior | 0.962 (0.918-0.984) |  | 0.892 (0.670-0.967) |
|  | 8. Mid anteroseptal | 0.974 (0.949-0.995) |  | 0.884 (0.648-0.965) |
|  | 9. Mid inferoseptal | 0.967 (0.802-0.991) |  | 0.781 (0.404-0.931) |
|  | 10. Mid inferior | 0.976 (0.956-0.984) |  | 0.835 (0.525-0.949) |
|  | 11. Mid inferolateral | 0.984 (0.951-0.995) |  | 0.818 (0.487-0.944) |
|  | 12. Mid anterolateral | 0.964 (0.933-0.980) |  | 0.917 (0.739-0.975) |
|  | 13. Apical anterior | 0.983 (0.966-0.988) |  | 0.849 (0.560-0.954) |
|  | 14. Apical septal | 0.980 (0.957-0.987) |  | 0.678 (0.203-0.895) |
|  | 15. Apical inferior | 0.983 (0.941-0.995) |  | 0.893 (0.674-0.968) |
|  | 16. Apical lateral | 0.974 (0.921-0.992) |  | 0.918 (0.743-0.976) |
|  |  |  |  |  |
| LS | 1. Basal anterior | 0.961 (0.883-0.987) |  | 0.783 (0.432-0.928) |
|  | 3. Basal inferoseptal | 0.709 (0.310-0.896) |  | 0.560 (0.042-0.841) |
|  | 4. Basal inferior | 0.724 (0.401-0.904) |  | 0.537 (-0.122-0.785) |
|  | 6. Basal anterolateral | 0.898 (0.715-0.966) |  | 0.918 (0.811-0.981) |
|  | 7. Mid anterior | 0.842 (0.580-0.946) |  | 0.782 (0.429-0.928) |
|  | 9. Mid inferoseptal | 0.704 (0.301-0.894) |  | 0.619 (0.132-0.865) |
|  | 10. Mid inferior | 0.816 (0.522-0.937) |  | 0.695 (0.261-0.896) |
|  | 12. Mid anterolateral | 0.926 (0.787-0.975) |  | 0.860 (0.606-0.955) |
|  | 13. Apical anterior | 0.920 (0.771-0.973) |  | 0.674 (0.223-0.887) |
|  | 14. Apical septal | 0.782 (0.449-0.924) |  | 0.574 (0.063-0.847) |
|  | 15. Apical inferior | 0.860 (0.622-0.953) |  | 0.555 (0.034-0.839) |
|  | 16. Apical lateral | 0.915 (0.759-0.972) |  | 0.857 (0.042-0.841) |
|  |  |  |  |  |
| SR-CS | 1. Basal anterior | 0.715 (0.509-0.922) |  | 0.721 (0.515-0.928) |
|  | 2. Basal anteroseptal | 0.919 (0.842-0.984) |  | 0.821 (0.744-0.916) |
|  | 3. Basal inferoseptal | 0.456 (0.153-0.751) |  | 0.444 (0.141-0.739) |
|  | 4. Basal inferior | 0.554 (0.253-0.859) |  | 0.504 (0.203-0.809) |
|  | 5. Basal inferolateral | 0.771 (0.611-0.938) |  | 0.561 (0.045-0.830) |
|  | 6. Basal anterolateral | 0.799 (0.661-0.913) |  | 0.761 (0.601-0.928) |
|  | 7. Mid anterior | 0.838 (0.735-0.942) |  | 0.812 (0.709-0.916) |
|  | 8. Mid anteroseptal | 0.773 (0.648-0.903) |  | 0.723 (0.598-0.853) |
|  | 9. Mid inferoseptal | 0.559 (0.262-0.839) |  | 0.534 (0.237-0.814) |
|  | 10. Mid inferior | 0.770 (0.631-0.915) |  | 0.741 (0.602-0.886) |
|  | 11. Mid inferolateral | 0.910 (0.850-0.975) |  | 0.876 (0.816-0.941) |
|  | 12. Mid anterolateral | 0.699 (0.491-0.894) |  | 0.651 (0.443-0.846) |
|  | 13. Apical anterior | 0.960 (0.931-0.994) |  | 0.949 (0.920-0.983) |
|  | 14. Apical septal | 0.855 (0.78-0.929) |  | 0.814 (0.739-0.888) |
|  | 15. Apical inferior | 0.817 (0.693-0.920) |  | 0.771 (0.647-0.874) |
|  | 16. Apical lateral | 0.928 (0.865-0.970) |  | 0.903 (0.840-0.945) |
|  |  |  |  |  |
| SR-LS | 1. Basal anterior | 0.697 (0.492-0.902) |  | 0.703 (0.498-0.908) |
|  | 3. Basal inferoseptal | 0.663 (0.433-0.893) |  | 0.442 (0.135-0.752) |
|  | 4. Basal inferior | 0.592 (0.292-0.892) |  | 0.342 (0.042-0.642) |
|  | 6. Basal anterolateral | 0.746 (0.5760.916) |  | 0.651 (0.421-0.881) |
|  | 7. Mid anterior | 0.792 (0.642-0.942) |  | 0.766 (0.616-0.916) |
|  | 9. Mid inferoseptal | 0.628 (0.363-0.893) |  | 0.531 (0.226-0.836) |
|  | 10. Mid inferior | 0.675 (0.475-0.875) |  | 0.579 (0.314-0.844) |
|  | 12. Mid anterolateral | 0.643 (0.393-0.893) |  | 0.595 (0.345-0.845) |
|  | 13. Apical anterior | 0.926 (0.886-0.966) |  | 0.915 (0.875-0.955) |
|  | 14. Apical septal | 0.790 (0.625-0.925) |  | 0.641 (0.441-0.841) |
|  | 15. Apical inferior | 0.824 (0.684-0.964) |  | 0.778 (0.638-0.918) |
|  | 16. Apical lateral | 0.880 (0.810-0.950) |  | 0.855 (0.785-0.925) |
|  |  |  |  |  |

Note. CS: circumferential strain, LS: longitudinal strain, SR: strain rate, CCT-FT: cardiac computed tomography feature tracking, CMR-FT: cardiac magnetic resonance feature tracking, ICC: intraclass correlation coefficient, CI: confidence intervals.
